# The Use of Functional Data Analysis to Evaluate Activity in a Spontaneous Model of Degenerative Joint Disease Associated Pain in Cats

**DOI:** 10.1371/journal.pone.0169576

**Published:** 2017-01-18

**Authors:** Margaret E. Gruen, Marcela Alfaro-Córdoba, Andrea E. Thomson, Alicia C. Worth, Ana-Maria Staicu, B. Duncan X. Lascelles

**Affiliations:** 1 Comparative Pain Research Program, Department of Clinical Sciences, College of Veterinary Medicine, North Carolina State University, Raleigh, North Carolina, United States of America; 2 Comparative Medicine Institute, North Carolina State University, Raleigh, North Carolina, United States of America; 3 Department of Statistics, North Carolina State University, Raleigh, North Carolina, United States of America; 4 Center for Pain Research and Innovation, University of North Carolina School of Dentistry, Chapel Hill, North Carolina, United States of America; Indiana University, UNITED STATES

## Abstract

**Introduction and objectives:**

Accelerometry is used as an objective measure of physical activity in humans and veterinary species. In cats, one important use of accelerometry is in the study of therapeutics designed to treat degenerative joint disease (DJD) associated pain, where it serves as the most widely applied objective outcome measure. These analyses have commonly used summary measures, calculating the mean activity per-minute over days and comparing between treatment periods. While this technique has been effective, information about the pattern of activity in cats is lost. In this study, functional data analysis was applied to activity data from client-owned cats with (n = 83) and without (n = 15) DJD. Functional data analysis retains information about the pattern of activity over the 24-hour day, providing insight into activity over time. We hypothesized that 1) cats without DJD would have higher activity counts and intensity of activity than cats with DJD; 2) that activity counts and intensity of activity in cats with DJD would be inversely correlated with total radiographic DJD burden and total orthopedic pain score; and 3) that activity counts and intensity would have a different pattern on weekends versus weekdays.

**Results and conclusions:**

Results showed marked inter-cat variability in activity. Cats exhibited a bimodal pattern of activity with a sharp peak in the morning and broader peak in the evening. Results further showed that this pattern was different on weekends than weekdays, with the morning peak being shifted to the right (later). Cats with DJD showed different patterns of activity from cats without DJD, though activity and intensity were not always lower; instead both the peaks and troughs of activity were less extreme than those of the cats without DJD. Functional data analysis provides insight into the pattern of activity in cats, and an alternative method for analyzing accelerometry data that incorporates fluctuations in activity across the day.

## Introduction

Physical activity is commonly affected in patients with degenerative joint diseases (DJD), and changes in activity may be used as an outcome measure for patients with arthritis and DJDs [1–4]. Self-reported activity is often inadequate for quantifying actual physical activity [5], as self-report is subject to biases due to recall and social desirability, and patients are likely to under- or over-estimate true activity. The advent of accelerometer based systems allows for objective assessment of activity, and these systems have allowed researchers to more specifically define activity patterns, as well as track changes in activity related to health statuses and response to interventions.

‘Activity’ monitors (accelerometers) measure changes in acceleration by detecting low-frequency accelerations sampled at frequencies that vary by device but are often high sub-second frequencies. These changes in acceleration are recorded by the device and are converted to ‘counts’ for a given epoch length of seconds to minutes. The counts are unit-less, and are generated by a voltage signal which is proportional to the individual unit’s measure (e.g. duration and/or intensity) of change in acceleration [6]. That is, these counts will be higher with higher magnitude of acceleration for a given epoch. Accelerometers may be classified by the number of axes in which they measure acceleration (uni-, bi-, or tri-axial), or may be omni-directional. These monitors have been evaluated in humans as measures of physical activity by comparison of activity counts against oxygen consumption, measured via indirect calorimetry [7] or doubly-labeled water.

There has been great interest in the ability to objectively measure physical activity since correlation coefficients between self-report and accelerometer-based measures are frequently low [8]. In humans, accelerometer-based activity monitoring has been used to assess activity during sleep [9], following stroke [10], and as a criterion for validation of physical activity survey/assessment tools [11]. Population based epidemiologic studies have used accelerometry to evaluate physical activity in people with arthritis [3, 12–14] while intervention studies have quantified effects of treatments on activity with some showing improvement [15] and others showing more equivocal effects [16]. Analytical methods across studies have varied resulting in calls for greater uniformity in the interpretation of accelerometer-based data [17, 18].

The establishment of criteria for defining sedentary behavior and high-activity behavior has not been standardized. Activity counts per-minute have been used to determine whether an individual is moving or sedentary, and sustained high levels of activity counts used to indicate high-intensity activity, however different studies have employed cut-off levels that lead to disparate conclusions. The effects of varied cut-offs were examined in a modeling experiment done by Masse et al. [18], where application of four algorithms for accelerometer-based data reduction resulted in marked differences in outcomes. Additional issues include the mismatches between the detection abilities of particular activity monitors in relation to the activity being studied (e.g. uni-axial, vertically sensitive accelerometers used to detect activity involved in riding a bicycle) [19], differences in output when worn on the hip vs. the wrist [20], and varying criteria for establishing length of wear. Frequently, algorithm based or visual inspection of data is used to determine estimated wear time. Despite these limitations, accelerometry remains the primary means of objective physical activity monitoring in patient populations.

In veterinary medicine, accelerometers have been used in studies involving many species. In dogs [21] and in cats [22], accelerometry, with specific accelerometer types, has been validated in a lab environment as a surrogate measure of distance moved. In both dogs and cats, accelerometer outputs (counts) were compared against objectively assessed distance moved using standardized software (Noldus^®^ Ethovision) designed for quantification of behavior [21, 22]. Validation studies were followed with feasibility studies to evaluate the ability of dogs and cats to tolerate wearing such monitors in their home environments. Interestingly, a study in dogs found that activity counts were higher on weekends as opposed to weekdays [23], while a study in cats found the opposite [22]. In the cat study, the subjects were laboratory-housed cats, and the lower activity counts seen during the weekend were attributed to the lower amount of human/caretaker activity in the facility during those days. This suggests that owner patterns of activity are likely important mediators of activity in pet dogs and cats, and that activity patterns may not be uniform across the week.

The most common application of accelerometry in veterinary medicine has been the study of spontaneous activity in dogs and cats with DJD/osteoarthritis (DJD/OA). Several studies have used accelerometers to measure activity in dogs [21, 24] and cats [25–27]. Moreover, both dogs [28] and cats with DJD/OA will show improvements in activity with analgesics, and motor activity has been used as an important objective outcome measure for analgesic treatments in cats with DJD/OA in multiple studies [25, 29–32].

In addition, there is recent increased interest in domestic dogs and cats as models of naturally-occurring DJDs in humans [33–35] as both species develop spontaneous disease with significant overlapping features with the human condition [36–38] including mobility impairment. Indoor cats, in particular, are intriguing as a model of spontaneous activity, as their daily activity is less confounded by human intervention (i.e. though influenced by human activity, their activity over the day is not dependent on whether or not they are taken for a walk). Thus far, the analysis of the activity data generated by accelerometers in cats has been fairly coarse and a better understanding of activity patterns or profiles, and the most useful approaches for analyzing activity data will benefit both our ability to interpret the effects of DJD/OA on activity in cats, and the applicability of this naturally-occurring model to translational research.

To date, accelerometry has been used in cats to describe normal activity under different feeding and housing conditions [39–41] and activity in response to weight management strategies [42, 43], in addition to the studies of analgesic treatments for DJD/OA and associated pain [29–32]. Across these studies, statistical analyses of activity data have varied widely in method, using a diversity of analytic designs generally based upon condensed data. Particularly in the drug intervention studies, cats may wear activity monitors for days to weeks. Considering that each 24-hour day may contain 1440 individual per-minute “counts,” these studies generate large volumes of data for analysis. Current analytic methods frequently collapse the data down to single summary values (e.g. total counts or average per-minute counts) for particular time spans, and information about the pattern of activity in cats is lost. Inter-cat variability in these summary measures, even within a housing condition, is high, making between group analyses difficult. This variability, coupled with a lack of knowledge of the most important metric to investigate, hinders the ability to fully understand the impact of disease and the effects of interventions on cat activity.

Functional data analysis (FDA) provides methods for analyzing data that are believed to arise from curves evaluated at a finite grid of points [44]. In particular, it allows for the use of the entire profile of daily activity counts (over a 24-hour day), rather than summary values. As a result, FDA allows analysis of data patterns without losing the richness of the information contained in the minute-by-minute counts [45]. A common technique in FDA is functional principal components analysis (FPCA), which examines the dominant modes of variation of the data as a method for understanding the major sources of data variability [45]. Functional data analysis has been applied to accelerometer data in recent studies of people [46–49], but to our knowledge has not been applied in the field of pain research or with data gathered from veterinary species.

Application of FDA to accelerometer data from cats offers an opportunity to examine the pattern of activity in cats, including potential identification of peaks of activity and quantification of the variability of activity, and the effects of covariates that vary over time [48]. FDA allows activity data from cats to be represented in new ways than have previously been described, and can aid in the detection of patterns or variations among the data [45] as well as inform decisions about the use of such data in evaluating therapeutics.

The objectives of this study were to use FDA to evaluate activity patterns and activity intensity in cats with and without DJD in order to better understand normal population distributions for each group. We hypothesized that 1) normal cats would have higher daily activity counts and intensity than cats with DJD; 2) daily activity counts and intensity in cats with DJD would be inversely correlated with total radiographic DJD burden and total orthopedic pain score; and 3) daily activity counts and intensity would have a different pattern on weekends vs. weekdays. To our knowledge, no studies have been published that examine activity patterns in well phenotyped cats (with and without DJD) in their home setting.

## Materials and Methods

### Subjects

Potential study subjects were identified from local primary care veterinarians or were self-referred by owners in response to advertisements for one of four clinical trials. The first trial was designed to investigate activity in normal cats (i.e. those without DJD) (previously unpublished data). Two other trials included in the exploratory analyses [25, 30] were designed to evaluate outcome measures and efficacy for a non-steroidal anti-inflammatory medication in cats with DJD and owner-rated mobility impairment. All trials were carried out with approval by the North Carolina State University College of Veterinary Medicine’s Institutional Animal Care and Use Committee (Protocols 11-102-O, 08-124-O).

### Inclusion and exclusion criteria

Inclusion criteria for the normal cat study included age over one year, weight over one kilogram (kg), and the absence of owner-rated mobility impairment. Inclusion criteria for the intervention trials have been previously described [25, 30, 50]. Briefly, cats were required to be greater than one year of age and weigh more than one kg, and to have a qualifying degree of owner-rated mobility impairment, joint pain on orthopedic examination, and radiographic evidence of DJD.

Exclusion criteria, common across trials, have been described previously [25, 30, 50] and included the presence of suspected or diagnosed infectious diseases, symptomatic cardiac disease, immune-mediated disease, neoplasia, inflammatory bowel disease, urinary tract infection, hyperthyroidism, and diabetes mellitus. Cats with stable chronic kidney disease (CKD) up to and including IRIS stage two [51] were eligible to enroll following demonstration of stable serum biochemistry and urinalysis results. Importantly, all cats were required to be indoor only and able to wear a collar, though they did not need to have a collar at the time of enrollment.

Recruited cats were examined by a veterinarian and received full physical, orthopedic, and neurologic examinations. Demographic data including age, weight (kg), and body condition score ([BCS] on a 9-point scale [52]) were recorded. Cats meeting eligibility criteria were then sedated, and orthogonal radiographs were made of each joint. Radiographs were reviewed for the presence of DJD/OA as described in [53] by a board-certified veterinary radiologist masked to the results of the orthopedic examination.

### Total pain scores

During the orthopedic examination, each joint and axial skeletal segment was palpated and manipulated to evaluate for signs of pain and instability. Responses for each joint or segment were scored using a previously published scale [53] where 0 = no resentment; 1 = mild withdrawal, mild resistance to manipulation; 2 = moderate withdrawal, body tenses, may orient to site, may vocalize/increase vocalization; 3 = orients to site, forcible withdrawal from manipulation, may vocalize or hiss or bite; 4 = tries to escape or prevent manipulation, bites or hisses, marked guarding of area. The scores for each individual joint or axial skeletal segment were summed to generate a total pain (TPain) score for each cat (possible range: 0–80). Based on scores from a previously described study of the prevalence of DJD in cats [53], TPain scores were further categorized as 0–2 = negligible/normal (as long as no single joint received a score of 2); 2–4 = low (a score of 2 was placed in this category if a single joint received a score of 2); 5–9 = moderate; ≥10 = high.

### Total DJD scores

Radiographs were evaluated and scored as previously described [53]. Briefly, each joint was evaluated for the presence and severity of radiographic changes indicative of DJD and scored on a scale from 0 (normal) to 10 (ankylosis) by a single investigator (BDXL). The scores for each individual joint or axial skeletal segment were summed to generate a total DJD (TDJD) score for each cat (possible range: 0–200). Again based on scores from the previously described study [53], TDJD scores were further categorized as 0–3 = negligible/normal; 4–12 = low; 13–24 = moderate; ≥25 = high.

### Activity monitors

**Fig 1:**
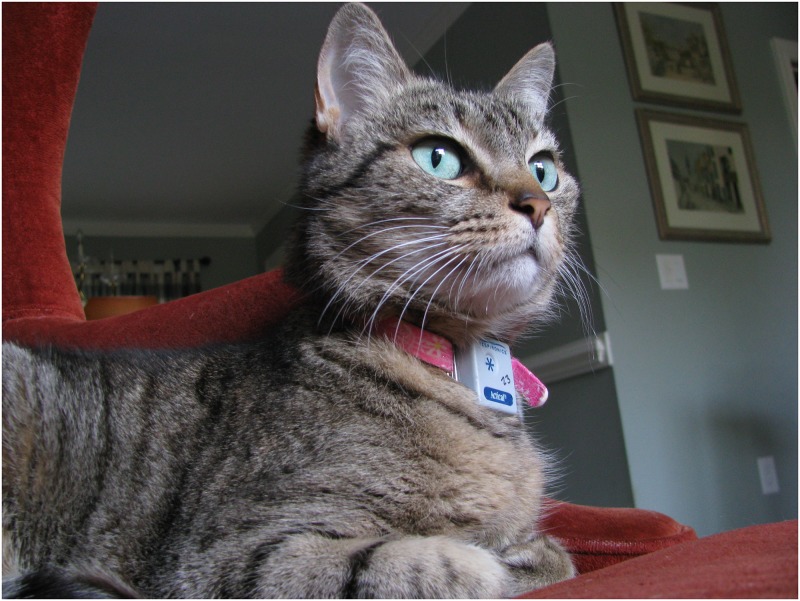
Cat wearing accelerometer. Client-owned cat wearing a collar-mounted Actical^®^ accelerometer in the typical position on the neck.

**Fig 2:**
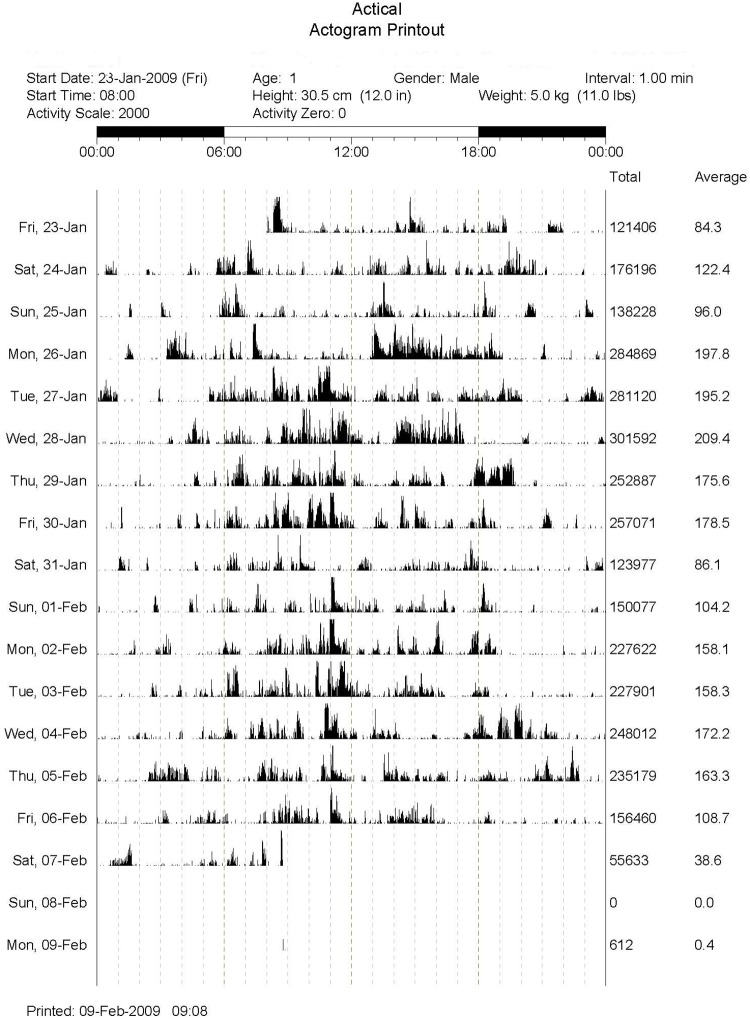
Example of an actigram for a cat. Each row of data contains the activity counts for a single day, with the counts depicted graphically along the time axis from 00:00 (12:00am) to 23:59 (11:59pm). The final two columns for each row represent the total activity counts for the day, and the average per-minute counts for the day.

Following enrollment, all cats in each study were fitted with an activity monitor (Actical^®^, Philips Respironics, Bend, Oregon, USA) mounted on a neck collar (Fig 1). Collars were provided by the study if the cats did not have their own. The Actical^®^ monitors are omni-directional activity monitors that contain a piezoelectric sensor mounted to an internal circuit board to generate analog voltage change that is proportional to the duration and intensity of the change in acceleration [6]. The Actical^®^ monitors have a sampling rate of 32 Hz, and report data (“counts”) for specified epoch lengths ranging from 15 seconds to one minute. Epoch length for summary data output by the units in these studies were set to 1 minute, and the collars were worn continuously throughout the study period, with the exception of periodic downloading. Each daily activity profile is thus composed of 1440 minute-by-minute measurements. Activity data were downloaded to a dedicated computer via a serial port reader (Actireader^®^) using designated software. The software generates a graphical representation of the activity over each day (Fig 2) as well as a ‘raw’ output of activity counts per-minute that can be exported into a spreadsheet for analysis. Cats in the normal activity group wore their collars for 15 days, and had 13 days of useable data (the initial and final day were deleted as cats had a variable number of hours with the collar on for these two days). Cats in the intervention studies wore their collars throughout their studies, and had 13 comparable baseline days (deleting the first day and the day the collars were brought in for download). In order to decrease variability due to initial collar acceptance, the last 7 full days of baseline were used in these analyses.

### Statistical analysis

#### Data disclosure

Portions of the data used for this manuscript have been previously published, however the current use and analysis represent original work. The activity data for the cats with DJD have appeared in two separate publications using a very simplistic approach to analysis [25, 30].

#### Data sets

The normal cat data set included cats that had no owner-noted mobility impairment, normal classifications for TDJD score and TPain score, and comprised data from n = 15 cats. Cats in the DJD data set had a combination of owner-noted mobility impairment and abnormal TDJD and TPain scores (n = 83). These cats represent populations from two individual studies referred to by their in-house names as FMPI (n = 25) and Low-Dose (n = 58).

#### Data analysis

Prior to the current FDA-based approaches, descriptive statistics were generated for demographic information for the cats, and compared using one-way ANOVA for continuous variables (age, BCS, TPain score and TDJD score) and Chi-squared testing for distribution of cat gender.

As activity counts were highly skewed, data were transformed using the equation *x*⟶ln(1 + *x*) and then averaged in each 5-minute interval to decrease variability [46]. For convenience, in the remainder of the paper we refer to the transformed data over the 5-minute intervals as activity counts. For all analyses, significance level was set at *α* = 0.05; when multiple tests were performed on the same data subset, a Bonferroni correction was applied as *α* = 0.05/(*k*) where *k* is the number of tests, and the adjusted p-value reported. Average activity was calculated for each group of cats and denoted as *Y*_*Gi*_*(t)* for the activity of cat *i* in group *G*. Further, a measure of intensity of activity was generated to control for a cat- or accelerometer-specific effect and referred to as *I*_*Gi*_*(t)*, for the intensity of activity of cat *i* in group *G*. Intensity provides a sense of the cat’s activity relative to its average activity, with higher intensity interpreted as more active than average. It was calculated using a cat’s average activity over the last seven days of the baseline period (*AveY*_*Gi*_*)* with the following equation:

$$I_{G_{i}}\left(t\right)=\;Y_{G_{i}}\left(t\right)-Ave(Y_{G_{i}}),$$

where *AveY*_*Gi*_ is the average across all seven days

#### Evaluation and comparison of activity profiles for normal cats and cats with DJD

We first needed to determine separation or pooling of data for weekend and weekday activity, and for the two groups of DJD cats. To characterize activity patterns in cats with and without DJD, average activity and intensity profiles were separated for weekends (Saturday and Sunday) and weekdays (Monday through Friday) within each group of cats. Likelihood ratio testing [54] was used to formally assess first whether weekend and weekday activity and intensity profiles were different for each group of cats, and second whether the activity and intensity profiles were different for the two groups of DJD cats (FMPI and Low-dose). For both the activity and intensity profiles of the two groups of DJD cats, the null hypothesis of no difference between the two groups’ means for weekends and weekdays was formally investigated in four main settings (where Bonferroni correction was applied): 1) no covariates; 2) controlling for age, BCS, and their interaction (age*BCS); 3) controlling for age, BCS, age*BCS interaction, and TPain score; and 4) controlling for age, BCS, age*BCS interaction, and TDJD score. Null distributions were based on N = 10,000 simulations. Further, we formally assessed whether the population distributions of activity and intensity profiles for the two groups of DJD cats were the same using the Anderson-Darling testing procedure proposed by Pomann et al. [55]. Results are discussed in the results section; they were supportive of separating weekend and weekday activity and pooling data from the two groups of cats with DJD into one DJD group.

To describe the main features of the average activity and intensity profiles of the cats, functional principal components analysis (FPCA) was performed for both the Normal cats and the combined group of cats with DJD (DJD group), separately for weekends and weekdays, with the following formula:

$$Y_{i}\left(t\right)=\;\mu \left(t\right)+\underset{k}{\sum^{\;}}\phi_{k}\left(t\right)\xi_{ik}+\epsilon_{it}$$

where *μ(t)* is the mean at each time point, *ϕ*_*k*_*(t)* is the *k* eigenfunction, and *ξ*_*ik*_ are the scores for the *k* component and *i* subject. To better understand the relationship between weekends and weekdays for each principal component, correlations were generated on the estimated scores for each of the *k* principal components.

Finally, to formally assess the effects of age, BCS, TDJD score, and TPain score on activity profiles and intensity profiles, we used functional regression models for the Normal and DJD groups separately for weekends and weekdays. Specifically, the assumed models for each response can be written for each group as specified in Faraway [56] and computed using Scheipl et al. [57] methods as follows:

Model 1: Normal cats

$$Y_{i}\left(t\right)=\beta_{0}\left(t\right)+\;Age_{i}\beta_{1}\left(t\right)+\;BCS_{i}\beta_{2}\left(t\right)+\;{\left(Age*BCS\right)}_{i}\beta_{3}\left(t\right)+\;\epsilon_{i}\left(t\right)$$

Model 2: DJD cats (where TDJD and TPain scores were included)

$$Y_{i}\left(t\right)=\beta_{0}\left(t\right)+\;Age_{i}\beta_{1}\left(t\right)+\;BCS_{i}\beta_{2}\left(t\right)+\;{\left(Age*BCS\right)}_{i}\beta_{3}\left(t\right)+\;TDJD_{i}\beta_{4}\left(t\right)+\;TPain_{i}\beta_{5}\left(t\right)+\;\epsilon_{i}\left(t\right)$$

where *β*_0_*(t)* is the intercept, *β*_1_*(t)* is the time-varying effect of age, *β*_2_*(t)* is the time-varying effect of BCS, *β*_3_*(t)* is the time-varying effect of the interaction between age and BCS, *β*_4_*(t)* is the time-varying effect of DJD score and *β*_5_*(t)* is the time varying effect of Pain score. Each covariate was standardized, and the coefficients *β*_0_*(t)*, *β*_1_*(t)*, *β*_2_*(t) and β*_3_*(t)* were modeled using penalized splines. Here *ϵ;*_*i*_*(t)* denotes the normal residual term, assumed to be independent, centered in zero with variance *σ*^2^ and identically distributed.

Finally, we compared the Normal group with the DJD group using both the daily activity and intensity profiles, separately for weekends and weekdays. Average activity profiles and intensity profiles for Normal cats and those with DJD were compared using the same analysis approach outlined for comparing the two groups of DJD cats for both group means and population distributions. Group means were formally investigated in the same four main settings, with and without covariates.

Statistical analysis was performed using the computing environment R (R Core Team, 2016). The code and data to perform each of the tests mentioned in this paper are available for download.

## Results

Descriptive statistics for the cats in each group are presented in Table 1. Cats in the Normal group were significantly younger and, as expected, had lower TDJD and TPain scores than cats in the FMPI and Low-dose groups. Cats in the FMPI and Low-dose groups were not significantly different for any of these variables.

**Table 1:** Demographic distribution for cats included in each of the studies. Results within a category that are designated by the same letter were not significantly different from one another.

Study	Mean Age (years)	Median Body Condition Score (1–9)	Sex (MC/FS)	Mean TDJD score (possible range:0–200)	Mean TPain score (possible range:0–80)
Normal cat (n = 15)	5.80—A	5	9/6	3.07—A	1.3—A
FMPI (n = 25)	11.77—B	7	8/17	20.0—B	14.0—B
Low-dose (n = 58)	12.4—B	6	27/31	23.4—B	16.4 –B,C
Between group analysis	ANOVA: p<0.001	Wilcoxon test: p = 0.060	Likelihood ratio: p = 0.360	ANOVA: p<0.0001	ANOVA: p<0.0001

Prior to transformation, range, quartiles, mean, and median average per-minute activity (across the 7 days) were calculated (Table 2). The range of activity counts for the normal cats is smaller than for the cats with DJD, however the mean average per-minute activity for each group is not significantly different (One way ANOVA, p = 0.541).

**Table 2:** Pre-transformation range, quartiles, median, and mean of average per-minute activity counts across the 7 day period for each group of cats.

Group	Minimum	1^st^ Quartile	Median	Mean	3^rd^ Quartile	Maximum
Normal	20.61	25.02	31.38	35.98	42.12	72.04
FMPI	11.44	22.09	30.87	39.70	54.92	105.70
Low-dose	8.22	21.31	31.42	34.97	41.05	108.00

### Evaluation of activity profiles for normal cats

**Fig 3:**
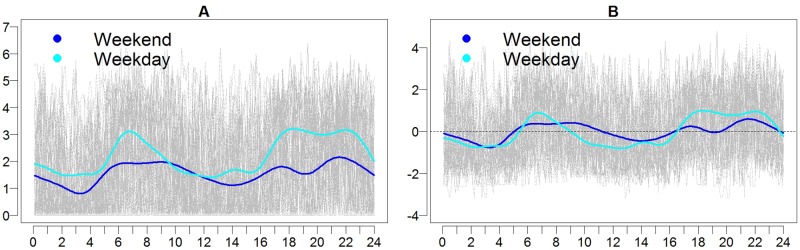
Activity (A) and intensity (B) profiles for cats in the Normal group. Log transformed activity for all cats is shown in gray with time (in hours) along the horizontal axis. The group mean for activity and intensity are shown for the weekends (dark blue) and weekdays (light blue). For intensity, positive values (above the zero line) indicate activity that is higher than average for that time period, while negative values (below the zero line) indicate activity that is lower than average.

Fig 3 depicts the average activity and intensity profiles for weekdays and weekends for the Normal cats. The times where the intensity is positive may be interpreted as times when the activity is greater than typical activity. Cats show a bimodal pattern of activity with a trough during the hours between approximately 2:00 am and 5:00 am. During the weekdays, the activity peaks occur in the morning between approximately 5:30 am and 9:00 am and in the evening between 17:00 (5:00 pm) and 23:00 (11:00 pm). This pattern is present but less well-defined on weekends, with the morning peak less extreme and shifted to the right. Likelihood ratio testing for activity and intensity tested the null hypothesis that mean weekend and weekday average activity and intensity were the same and obtained p-values of <0.0001 for each, indicating a statistically significant difference between weekends and weekdays for means of average activity and intensity profiles.

### Evaluation of activity profiles for DJD cats

The two sets of data from cats with DJD (FMPI and Low-dose studies) were evaluated for a difference in activity and intensity profiles prior to pooling. The test for a difference in the distributions showed no significant difference for the weekends (p = 0.114) or the weekdays (p = 0.139). Activity and intensity profiles from the two groups are shown in S1 Fig. Bonferroni corrected results of likelihood ratio tests showed no significant differences between the two sets of cats in models that included no covariates (p = 0.790 for activity and p = 0.986 for intensity), or controlled for age, BCS, the interaction between age and BCS, TPain score, and TDJD score (all p-values >0.050 for both activity and intensity). Given the lack of evidence of a difference between the groups for activity and intensity profiles, the two groups were pooled.

**Fig 4:**
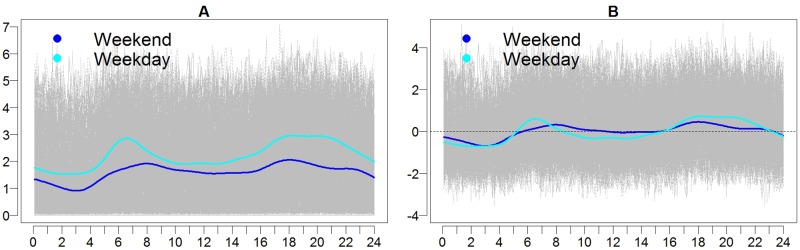
Activity (A) and intensity (B) profiles for cats in the DJD group. Log transformed activity for all cats is shown in gray with time (in hours) along the horizontal axis. The group means for activity and intensity are shown for weekends (dark blue) and weekdays (light blue).

Following pooling of the data, and as with the Normal group of cats, average activity and intensity profiles for DJD cats for weekends and weekdays were generated (Fig 4). Again, the bimodal pattern of activity was noted, with peaks in activity and intensity evident in the morning from approximately 5:00 am to 8:30 am, and evening from approximately 16:00 (4:00 pm) to 23:00 (11:00 pm). This was particularly apparent during weekdays and to a lesser extent during the weekends. Likelihood ratio tests for activity and intensity tested the null hypothesis that mean weekend and weekday average activity and intensity were the same, and obtained p-values of <0.001 for each, indicating a difference between weekends and weekdays for means of average activity and intensity.

**Fig 5:**
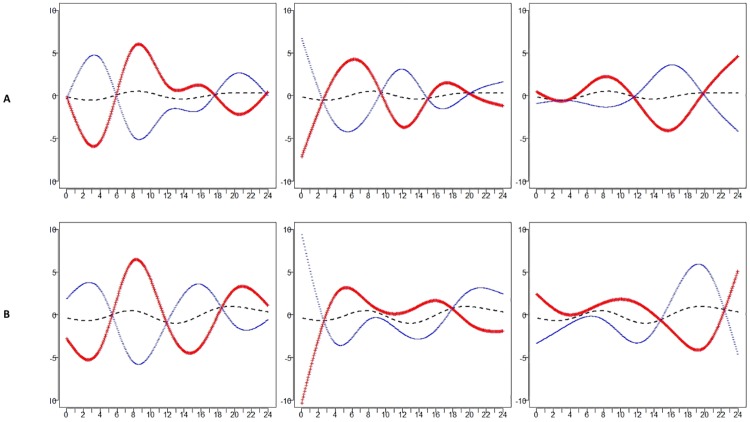
FPCA for Intensity for the Normal cats for weekends (A) and weekdays (B). Hours of the day are shown across the horizontal axis. Variance about the mean that corresponds to each FPC is shown for weekends and weekdays with red (pluses) indicating the positive direction and blue (minuses) indicating the negative direction. Variance explained by each FPC: Weekends: 35.88%, 24.44%, 16.83%. Weekdays: 40.01%, 23.86%, 21.77%.

**Fig 6:**
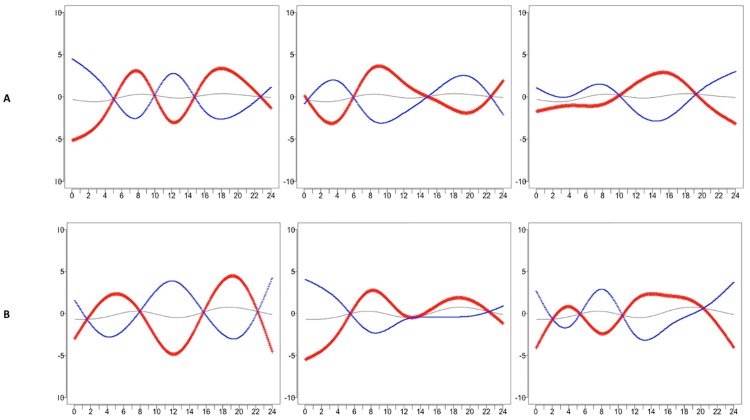
FPCA for Intensity for the DJD cats for weekends (A) and weekdays (B). Variance about the mean corresponding to each FPC is shown for weekends and weekdays with red (pluses) indicating the positive direction and blue (minuses) indicating the negative direction. Variance explained by each FPC: Weekends: 33.99%, 21.80%, 16.67%. Weekdays: 36.93%, 20.16%, 18.58%.

As weekend and weekday profiles have different distributions, FPCA was performed separately for weekend and weekday data, and results are presented here for intensity profiles for the Normal group and DJD group. The top three eigenfunctions are shown in S2 and S3 Figs. Figs 5 and 6 display the variation about the estimated mean corresponding to each direction: $\hat{\mu }\left(t\right)\pm 2\sqrt{\hat{\lambda_{k}}}\hat{\phi_{k}}\left(t\right)$, where $\hat{\lambda_{k}}$ is the estimated eigenvalue for each of the top three eigenfunctions *ϕ*_*k*_*(t)*,for the Normal group and DJD group, respectively.

For the Normal group, the three components explain approximately 77% and 86% of the total variance for weekend and weekday intensity, respectively. Correlations between the scores for weekends and weekdays were 0.77 for FPC1, 0.53 for FPC2, and 0.64 for FPC3. For both weekends and weekdays, the variance about the mean for FPC1 shows a sign change at the beginning and end of each peak, while FPC3 shows an important peak during the evening on the weekdays that is shifted earlier on weekends. Cats that are positively loaded on FPC1 show similar behavior on weekends and weekdays during the period from midnight to 6:00 am, but different behavior on weekends and weekdays during the period from noon to midnight.

For the DJD group, the three components explain approximately 73% and 77% of the total variance for weekend and weekday intensity, respectively. Correlations between the scores for weekends and weekdays were 0.38 for FPC1, 0.41 for FPC2, and 0.49 for FPC3. For both weekends and weekdays, the variance about the mean for FPC1 shows a sign change at the beginning and end of each peak. Cats that are positively loaded on FPC1 have lower than average activity during early mornings on weekdays, higher than average activity between 10:00 and 15:00 (3:00 pm), and lower again after 15:00 (3:00 pm), while a different pattern is seen for weekends. FPC2 and FPC3 also show different patterns for weekends and weekdays, particularly between 15:00 (3:00 pm) and midnight for PFC2 and midnight to 8:00 am for FPC3.

**Fig 7:**
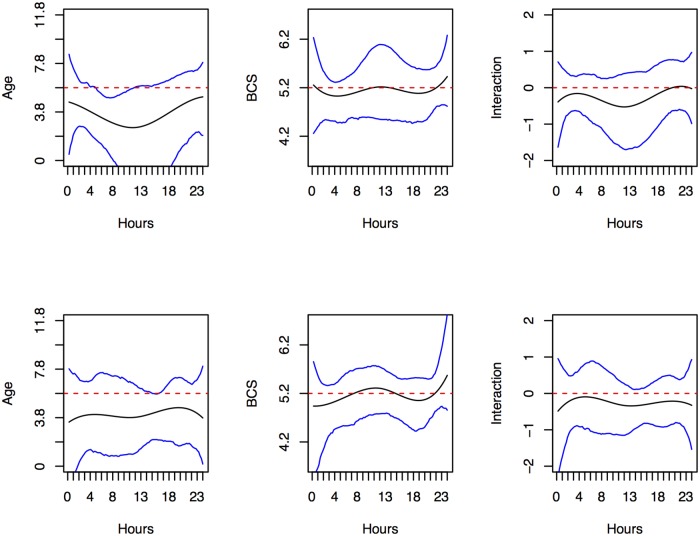
Functional regression analysis for activity in the Normal group. Depicted are the smooth effects of Age (in years, left panels), BCS (middle panels) and AGE*BCS (standard deviations away from the mean, right panels) on the activity of Normal cats, when model (1) is assumed. Results are shown for weekends in the top row and weekdays in the bottom row, with functional coefficients in black, 95% confidence intervals in blue, and zero demarcated in red.

Results of functional regression analysis evaluating the effects of age and BCS on activity and intensity profiles for weekends and weekdays in the Normal group are shown in Fig 7 (activity) and S4 Fig (intensity). Specifically, each panel depicts the estimated effect of age, BCS and their interaction in the solid line, as well as their 95% point-wise confidence intervals (CIs) constructed using bootstrap methods (N = 2000). Results are significant when the bounds of both CIs are above or below zero; no significant effects were found for intensity profiles for weekends or weekdays. For activity profiles, age, was significantly associated, though the pattern differed over time. Older cats were more likely to be less active in the mornings on weekends, and the afternoons on weekdays.

**Fig 8:**
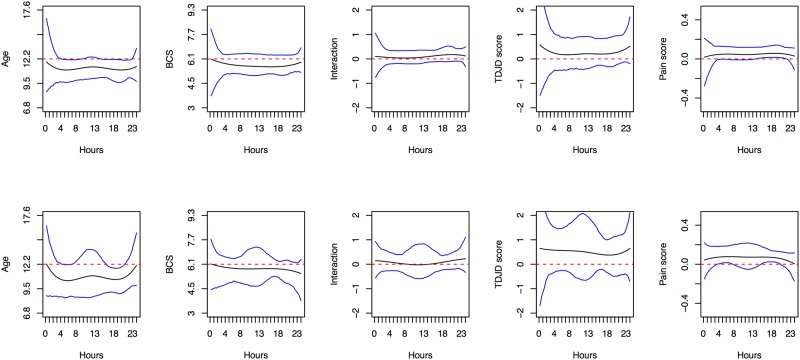
Functional regression analysis for activity in the DJD group. Depicted are the smooth effects of Age (in years), BCS, AGE*BCS (standard deviations away from the mean), standardized TDJD score and TPain score on the activity of DJD cats, when model (2) is assumed. Results are shown for weekends in the top row and weekdays in the bottom row, with functional coefficients in black, 95% confidence intervals in blue, and zero demarcated in red.

Results of functional regression analysis evaluating the effects of age, BCS, TDJD score, and TPain score on activity and intensity for weekends and weekdays in the DJD cats are shown in Fig 8 (activity) and S5 Fig (intensity). Specifically, each panel depicts the estimated effect of age, BCS and their interaction, TDJD score, and TPain score in solid line, as well as their 95% point-wise CIs constructed using bootstrap methods. Again, results are significant when the bounds of both CIs are above or below zero; no significant effects were found for intensity for weekends or weekdays. For activity profiles, age and pain score were significantly associated, though the pattern differed over time. During the morning and afternoon peaks, older cats were more likely to be less active on both weekends and weekdays. However, cats with higher TPain score were more likely to be more active during the daytime hours on both weekends and weekdays.

### Comparison of activity profiles between normal cats and those with DJD

Likelihood ratio tests were used to assess the null hypothesis that activity profiles during weekends and weekdays were the same between the Normal cats and those in the DJD group. Separate tests were run with no covariates and controlling for covariates; p-values for results are summarized in Table 3 and show a difference in intensity profile between Normal cats and those with DJD during the weekdays, and a difference in activity profile between Normal cats and those with DJD on the weekends when controlling for covariates. The results indicate different mean intensity during weekdays for the two groups; for the other group comparisons, the differences in the way the responses vary seem to be more complex.

**Table 3:** Results of likelihood ratio tests for weekend and weekday activity profiles and intensity profiles when comparing Normal and DJD cats. Models were tested both with and without covariates. Values in bold are significant after adjustment for multiple comparisons using Bonferroni correction for each data set (row).

	No covariates	Age, BCS, Age*BCS interaction	Age, BCS, Age*BCS interaction, and TDJD score	Age, BCS, Age*BCS interaction, TPain score
Weekend *activity profile*	0.6648	**<0.0001**	**<0.0001**	0.0320
Weekend *intensity profile*	0.9098	0.9900	0.9900	0.9999
Weekday *activity profile*	0.9999	0.0292	0.0880	0.6736
Weekday *intensity profile*	**<0.0001**	**<0.0001**	**<0.0001**	**<0.0001**

**Fig 9:**
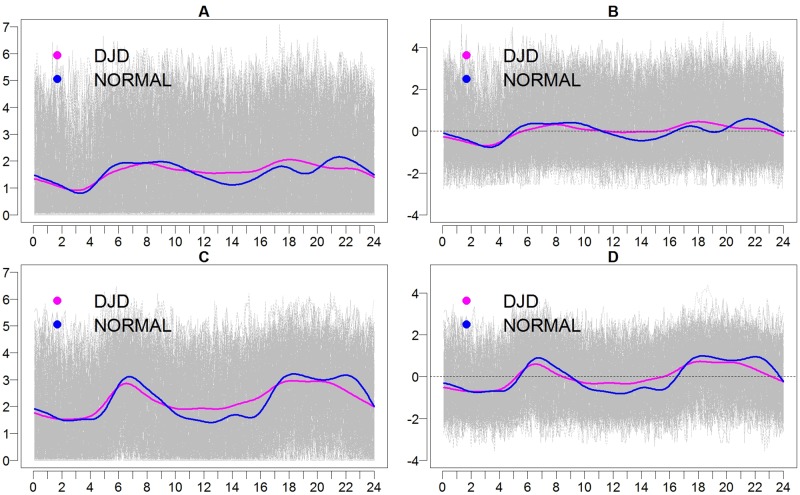
Activity (A and C) and intensity (B and D) profiles for cats in the Normal group (blue) and those with DJD (pink). Log transformed activity for all cats is shown in gray with time (in hours) along the horizontal axis. The group mean for activity and intensity are shown for weekends (A&B, respectively) and weekdays (C&D, respectively).

Finally, we investigated whether the way the activity varies (the population distributions) is the same in Normal cats and DJD cats; for this we used the functional Anderson-Darling testing procedure of Pomann et al. [55]. We found significant evidence against this null hypothesis for both weekends (p = 0.013) and weekdays (p = 0.010). The same null hypothesis was investigated for intensity and the results were also significant for both weekends (p<0.010) and weekdays (p<0.010) Based on these findings we conclude that the Normal cats and DJD cats show different levels of activity both during the weekends and weekdays, and also show different intensity of activity. This test indicates that the distributions are different, without providing information on how they are different. Visual inspection of the mean functions (Fig 9) shows that they cross each other at various times of the day, with the Normal cats having more variable activity and intensity, while the activity and intensity profile of the cats with DJD appears muted across the day.

## Discussion

In this study, FDA methods were used to examine the pattern of activity and intensity in cats with and without DJD. This represents a novel method of analysis, one that allows for further understanding of spontaneous activity profiles in cats, and how age, pain, and radiographic DJD affect activity profiles across the day. This understanding is critical to our ability to use activity as an effective objective outcome measure, both for monitoring an individual cat longitudinally, but also in response to a therapeutic intervention. As cats represent a spontaneous model of naturally-occurring DJD, further understanding of activity can expand our ability to investigate treatment options that may be of benefit to humans as well as cats. Through FDA, we now understand how DJD-associated pain alters the spontaneous activity profile, and points the way forward to how to assess the effects of analgesic treatments in a sophisticated and elegant manner, rather than looking at a coarse summary variable such as mean activity per-minute across a unit time period.

Using a group of normal cats, without DJD and associated pain, as well as cats with varying degrees of DJD, this study identified significant differences between the activity pattern of cats during the weekdays and weekends. These differences are important as they highlight a point raised by Piccione et al. regarding the influence of human activity on the activity pattern of cats [39, 40]. Cats are generally defined as diurnal or crepuscular, with peaks of activity at dawn and dusk [58, 59], a pattern that matches the bimodal pattern seen in this study. However, cats may readily adapt to different housing conditions. In an intriguing study done in 2013, Piccione et al. showed that cats that are kept outdoors overnight have a strikingly different pattern of activity, and an increase in overall activity, compared to cats that are kept indoors overnight [40]. The authors of that study proposed that human activity was the major influencing factor on the pattern of cat activity for cats housed exclusively indoors. The current study supports and extends these findings by showing that activity on weekends, when owners typically have an altered schedule, is different from weekdays. Here, the weekend activity across all groups of cats had more muted peaks, with the morning peak shifted to the right (later), suggesting that the morning activity began later on weekends. This could be explained by a strong influence of owner activity on cat activity. As owners are more likely to wake up and begin daily routines at more variable times on weekends, each cat’s peak of activity would be less uniformly distributed, contributing to the flattening of the peak. However, on weekdays, where owners are more likely to get up earlier and leave the house at more uniform times, activity related to caretaking (feeding, medication, play, etc.) is more likely to be concentrated in the morning. This may be less uniform in the evening, as owners return home at different times and may interact with their cat in a variety of ways over the hours between returning home and retiring to bed. This is supported by the narrower peak seen in the morning and the broader evening peak seen on weekdays. For the cats in these studies, demographic information collected from owners quantified how many hours owners were away from home, but not the details of when those hours occurred, making it difficult to explore whether there was a difference in the distribution of activity for cats whose owners were out of the home during working hours versus those that were not. Work is currently underway to explore this relationship by having a cohort of cats wear activity monitors while owners detail their times in and out of the home, as well as their interactions with their cats around food, play, and social interactions. While this should be further explored in studies that account for owner schedules, it suggests that when using activity data from cats, the number of weekend and weekdays should be standardized across data sets. This has also been suggested by studies in humans [60] and dogs [23].

The differential effect of weekdays and weekends on activity is particularly important to account for when evaluating treatment response. One study performed in laboratory cats [29] selected nighttime activity on weekends in order to decrease the effect of the human caretakers on spontaneous activity, thus attempting to focus on the activity modulating effects of the analgesic drugs being administered. This approach is interesting, and certainly decreases variability as cat and caretaker interaction can differ, but may underestimate the potential effect of the analgesic to increase activity in response to human interaction. If cat activity is heavily influenced by human activity, then hypothetically, analgesic treatment could lead to increased interest in interactions and thus increased activity.

In addition to the differences in activity profiles over weekends and weekdays, activity profiles and intensity profiles are different between Normal cats and those with DJD. However, it is not as simple as finding that the activity and intensity are consistently higher for the Normal cats as opposed to those with DJD. Indeed, a direct comparison of mean activity counts per minute in Normal cats versus cats with DJD showed no difference in activity. Nevertheless, when using FDA to evaluate activity patterns, we found that cats with DJD appear to have higher activity and intensity of activity at some times during the day, while the height of their peaks appears flattened compared to the Normal cats at other times. Overall, the variation in activity over the day appears to be muted, with lower peaks and less deep troughs, in cats with DJD compared to Normal cats. Cats typically experience bouts of activity in spurts rather than sustained trotting or running as might be seen in dogs. It is possible that the height of the peaks for the Normal cats represents relatively more of these bursts of activity, so that what may be important is the height and number of the peaks. In humans, age has been associated with an increase in low-intensity activity at the expense of high-intensity activity, with high-intensity physical training in older persons resulting in a compensatory decrease in low-intensity activity [61]. As age is associated with chronic pain, this compensatory relationship may be pain related.

Cats experiencing joint-related pain may show a similar decrease in the number of spurts of activity, while maintaining a more consistent level of low-intensity activity. Pain-induced restlessness could contribute to this low-intensity activity, and this deserves further investigation. While work has been done in dogs to establish cut-points for distinguishing intensity of activity [62], such work has not been done in cats. Age has previously been shown to be associated with decreased activity in both cats and dogs [23], and cut-points in accelerometer counts for defining intensity of activity may need to reflect changes in baseline activity that occur with age. In the present study, functional regression showed that in the DJD groups, older cats were more likely to be less active across the majority of the day on both weekends and weekdays, but specifically in the morning and afternoon. Also in the DJD groups, cats with higher pain scores on orthopedic exam were more likely to show increased activity during the morning and afternoon. The reason for this is unknown, but may be related to the incongruency between pain on veterinary orthopedic exam and decreased mobility/activity in the home. Results from the FPCA suggest that morning and evening peaks (the mean behavioral pattern) account for the majority of variability. The first dominant mode of variation for both Normal and DJD cats represents the morning and evening peaks as having a different pattern of variation from the rest of the day (during weekdays). This variation pattern explains almost half of the total variance (approximately 40% for each). While the FPCs for weekends and weekdays are not controlled for cats (i.e. cats may be loaded positively on FPC1 for weekends and negatively loaded for FPC1 on weekdays), their scores are positively correlated indicating that if a cat is positively loaded on a component for the weekend, they are likely to be positively loaded on that component for weekdays.

Additional areas of interest in understanding activity patterns in cats include 1) defining “normal” activity for a cat of a given age or health status, as this would be valuable for determining an individual’s status relative to a population norm for their age, and 2) being able to use baseline activity to stratify cats for randomization in clinical trials. Prior to log transformation of the data, ranges, means, and medians of average per-minute activity across the period were generated, and showed similarity between the means and medians for all groups of cats. While FDA showed that the mean activity profile over the day was different at times, it would not be possible to classify a cat as normal or abnormal based only on their activity counts. Therefore, the first goal does not appear possible; cats show variability in activity independent of DJD and pain, similar to variability seen in people, though additional studies with larger numbers of cats should be performed. However, using the median average per-minute activity, the second goal is potentially achievable. In general, studies of therapeutic interventions for DJD have randomized or stratified based on an owner rating [30] or radiographic DJD [29], and then used activity as the objective outcome measure. Median per-minute activity could be used as a variable for randomization to a clinical study group, or even as an entry criterion for early clinical studies, assuming that lower median activity indicates pain-related decrease in activity.

### Limitations and future work

This study was designed to evaluate differences in activity patterns between a group of Normal cats and a group of cats with DJD using functional data analysis. However, several limitations exist which warrant discussion. First, the group of Normal cats was smaller in number and significantly younger than the group of cats with DJD. In contrast, the cats with DJD were necessarily more impaired, as these were cats selected for inclusion in clinical trials, with a relatively high bar set for entry. While these groups of cats were not matched for age, and age is clearly important in physical activity level, it is difficult to find older cats without radiographic DJD (and associated pain) as prevalence of radiographic DJD in cats has been estimated at 60–92% of cats, with increased prevalence associated with age [63, 64]. The current study required cats that were classified as Normal to have minimal to no radiographic evidence of DJD. As radiographic disease does not correlate perfectly with the presence of pain [65], this study further required that cats defined as Normal have minimal to no pain on orthopedic exam. This qualification was required as it is not yet known what degree of pain on orthopedic exam or radiographic DJD corresponds to clinically relevant pain or mobility impairment. Indeed, a study by Guillot et al. [29] included a group of cats classified as having abnormal orthopedic exam findings but no radiographic evidence of OA, and these cats were not impaired on peak vertical force, a measure generally considered more sensitive than simple observation, suggesting that pain on orthopedic exam may not translate to clinical signs of impairment. Given the discrepancies between the groups of cats, future work to understand activity patterns in cats should use a randomly selected group of cats of varying ages and phenotype them following the collection of activity data. This would allow better understanding of whether there is a breakpoint for pain on exam or radiographic DJD that predicts lower activity.

Still, given the dichotomy of the two populations used in this study, it is even more striking that the activity patterns were not more distinct between the Normal and DJD cats. In cats, as in dogs and people, there exists a wide variation of activity levels. For clinical trials, it may be possible to randomize cats based on baseline activity, but the inter-cat variability and generally small number of cats enrolled in clinical trials suggest that cats will continue to need to be evaluated as their own controls for intervention trials. In this study, the Normal cats had a more restricted range of per-minute activity than any of the other groups of cats, but this could be due to the smaller number of cats in this group, and expansion of this group could show a wider range of per-minute activity, though frequently smaller numbers are associated with greater variability. While all activity monitors were worn in the same manner, mounted on a neck collar, the same set of activity monitors was not necessarily used in each study. While laboratory based validity calibration has been performed for activity monitors in cats [22], reliability calibration is not routinely performed outside of that provided by the manufacturer at intermittent times. Inter-accelerometer variability has been shown to be higher than intra-accelerometer variability [66], and unpublished data from our collaborator suggests that while the activity monitors are internally consistent, some may register activity counts at lower acceleration, resulting in higher activity counts. This may be accounted for in our use of intensity, which compensates not only for inter-cat variability, but also for uncalibrated accelerometers or varying output from accelerometers. Using intensity, this study did not show significant effects for covariates within a group, but did find significant differences between the Normal cats and those with DJD during the weekdays.

## Conclusions

This type of FDA is novel for activity data in companion animals. The similarities between the FMPI and Low-dose studies suggest that the differences found between the Normal cats and those with DJD are real differences, but this should be explored more in future work. In addition, future work should evaluate the change in activity pattern in response to an analgesic therapy. While several studies have shown that analgesics can increase activity in cats with DJD [25, 29], these studies have all used average per-minute activity over a treatment period. Functional data analysis can help us understand the pattern of these improvements. Based on the results of this study, we suggest that changes in activity in response to an analgesic might be most apparent during the morning and evening peak on the weekdays, when the activity of cats with DJD appears lower than that of cats without DJD, and has the opportunity to increase in response to interactions with owners. However, alternatives to this suggestion are possible, and this will be an area of future research.

Further work that incorporates owner schedules will increase the granularity of analyses, and can shed light onto the effect of owner presence on activity peaks and on increased activity in response to an analgesic. Indeed, a potential placebo-by-proxy effect could be explored using FDA, as activity in cats should increase more when their owners are home if this effect is beneficial. Of great benefit to the understanding of activity in cats would be a longitudinal study of cats over their lifetime, from youth through to advanced age, correlated with changes in pain, radiographic DJD, weight, and health status. This type of study should also include additional subjective assessments of cats, including observations of temperament and behavior. It is possible that the interaction between pain, radiographic DJD, and activity is complex, as is seen in humans, and that behavioral traits interact both with baseline activity as well as response to DJD and associated pain. Future work to better understand temperament traits in cats is ongoing, and incorporation of these findings into studies of activity and treatment response will deepen our interpretation of results.

## Supporting Information

S1 Fig Activity (A) and intensity (B) profiles for cats in the two DJD groups. Log transformed activity for all cats is shown in gray with time (in hours) along the horizontal axis. The group mean for activity and intensity are shown for the Low-dose study (red) and FMPI study (yellow).
(TIF)

S2 Fig Results of FPCA for Intensity for the Normal cats. (A) The top three eigenfunctions of intensity profiles for activity during the weekend (black) and weekday (blue). The first component describes approximately 40% of the total variance for both weekends and weekdays and picks up the two peaks seen in the activity profiles.
(TIF)

S3 Fig Results of FPCA for Intensity for the DJD cats. The top three eigenfunctions of intensity profiles for activity during the weekend (dark blue) and weekday (light blue). The first component describes approximately 34% of the total variance for the weekends and approximately 37% for the weekdays and picks up the two peaks seen in the activity profiles.
(TIF)

S4 Fig Functional regression analysis for intensity in the Normal group. Depicted are the smooth effects of Age (years, left panels), BCS (middle panels) and AGE*BCS (standard deviations away from the mean, right panels) on the intensity of Normal cats, when model (1) is assumed. Results are shown for weekends in the top row and weekdays in the bottom row, with functional coefficients in black, 95% confidence intervals in blue, and zero demarcated in red.
(TIFF)

S5 Fig Functional regression analysis for intensity in the DJD group. Depicted are the smooth effects of Age (years), BCS, AGE*BCS (standard deviations away from the mean), standardized TDJD score and TPain score on the intensity of DJD cats, when model (2) is assumed. Results are shown for weekends in the top row and weekdays in the bottom row, with functional coefficients in black, 95% confidence intervals in blue, and zero demarcated in red.
(TIFF)
