# Supplementary figures and images for: The Use of Functional Data Analysis to Evaluate Activity in a Spontaneous Model of Degenerative Joint Disease Associated Pain in Cats
Source: PLoS One. 2017 Jan 18;12(1):e0169576. doi: 10.1371/journal.pone.0169576 (PMC5242440; doi:10.1371/journal.pone.0169576)

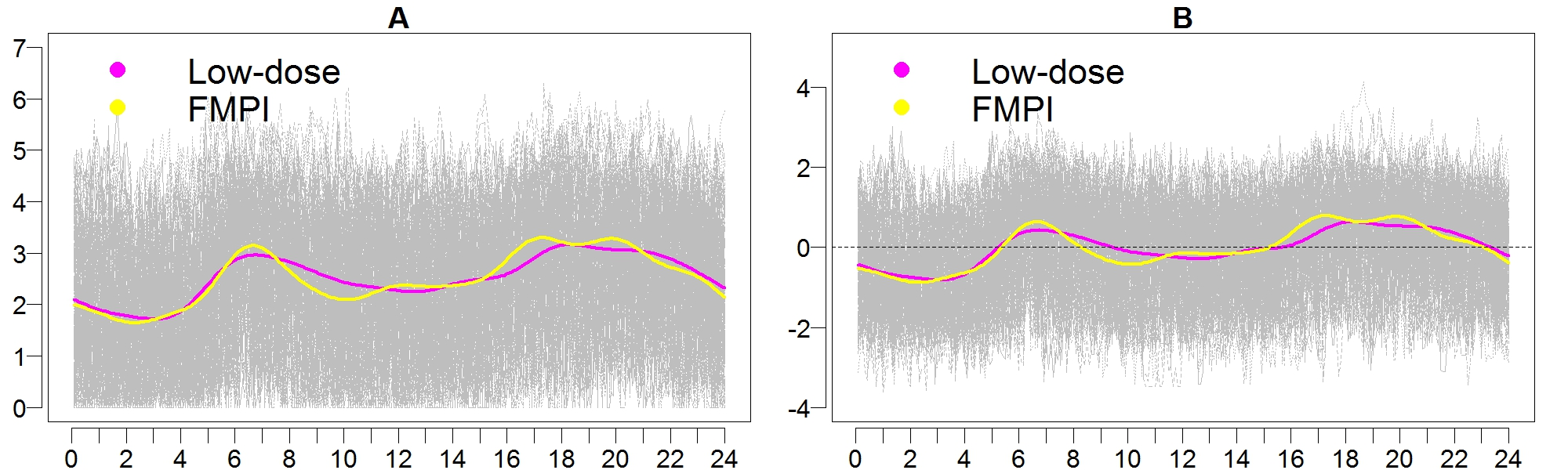

Supplement: S1 Fig — Log transformed activity for all cats is shown in gray with time (in hours) along the horizontal axis. The group mean for activity and intensity are shown for the Low-dose study (red) and FMPI study (yellow). (TIF) [file pone.0169576.s001.tif]

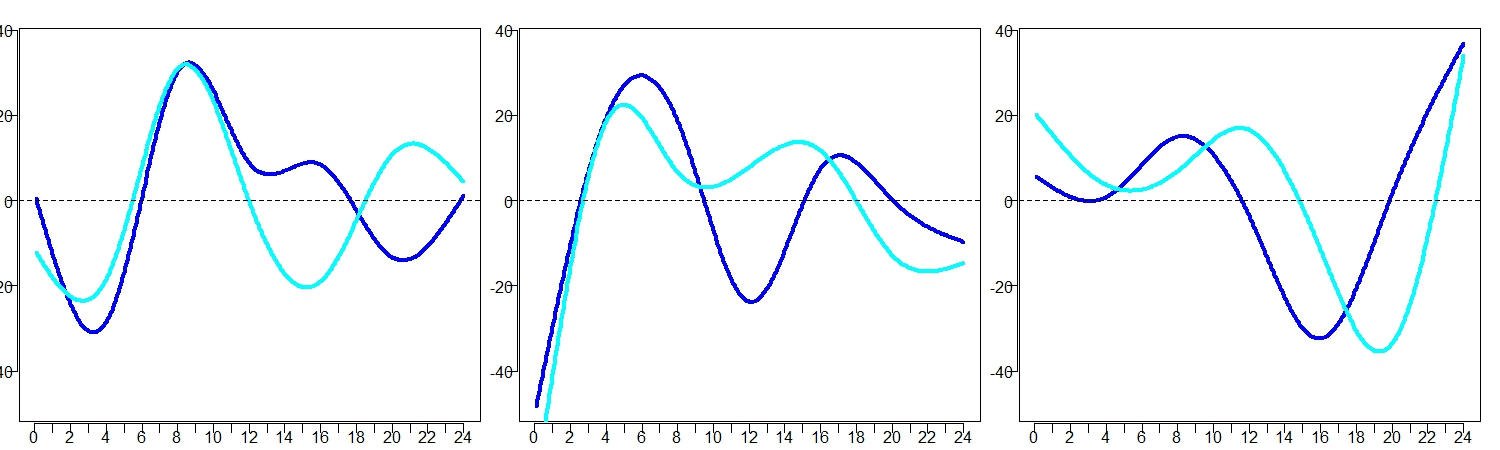

Supplement: S2 Fig — (A) The top three eigenfunctions of intensity profiles for activity during the weekend (black) and weekday (blue). The first component describes approximately 40% of the total variance for both weekends and weekdays and picks up the two peaks seen in the activity profiles. (TIF) [file pone.0169576.s002.tif]

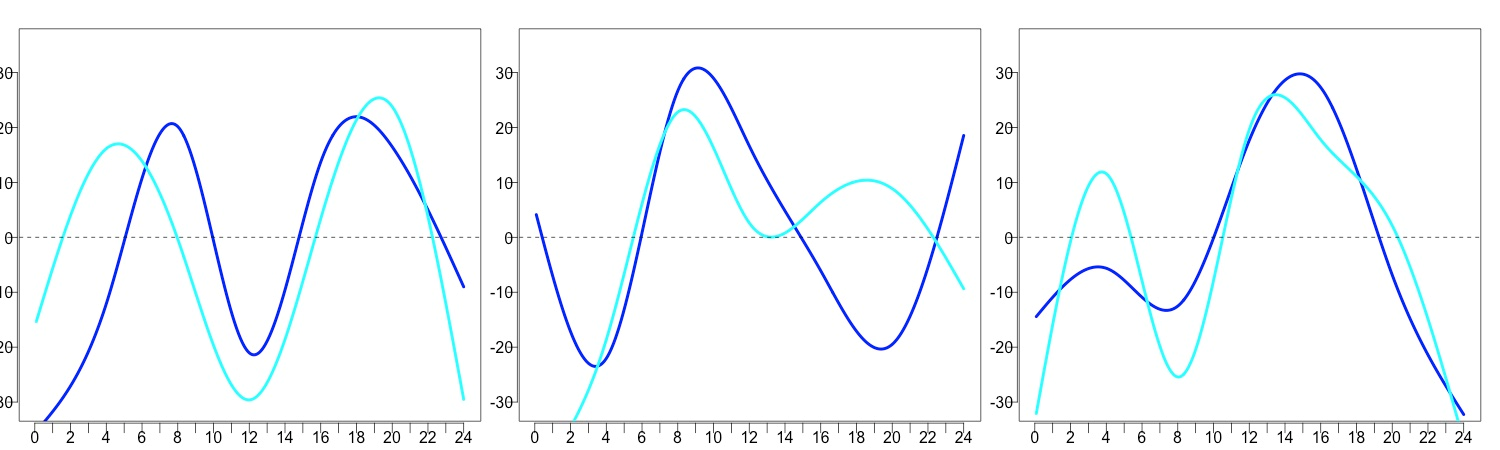

Supplement: S3 Fig — The top three eigenfunctions of intensity profiles for activity during the weekend (dark blue) and weekday (light blue). The first component describes approximately 34% of the total variance for the weekends and approximately 37% for the weekdays and picks up the two peaks seen in the activity profiles. (TIF) [file pone.0169576.s003.tif]

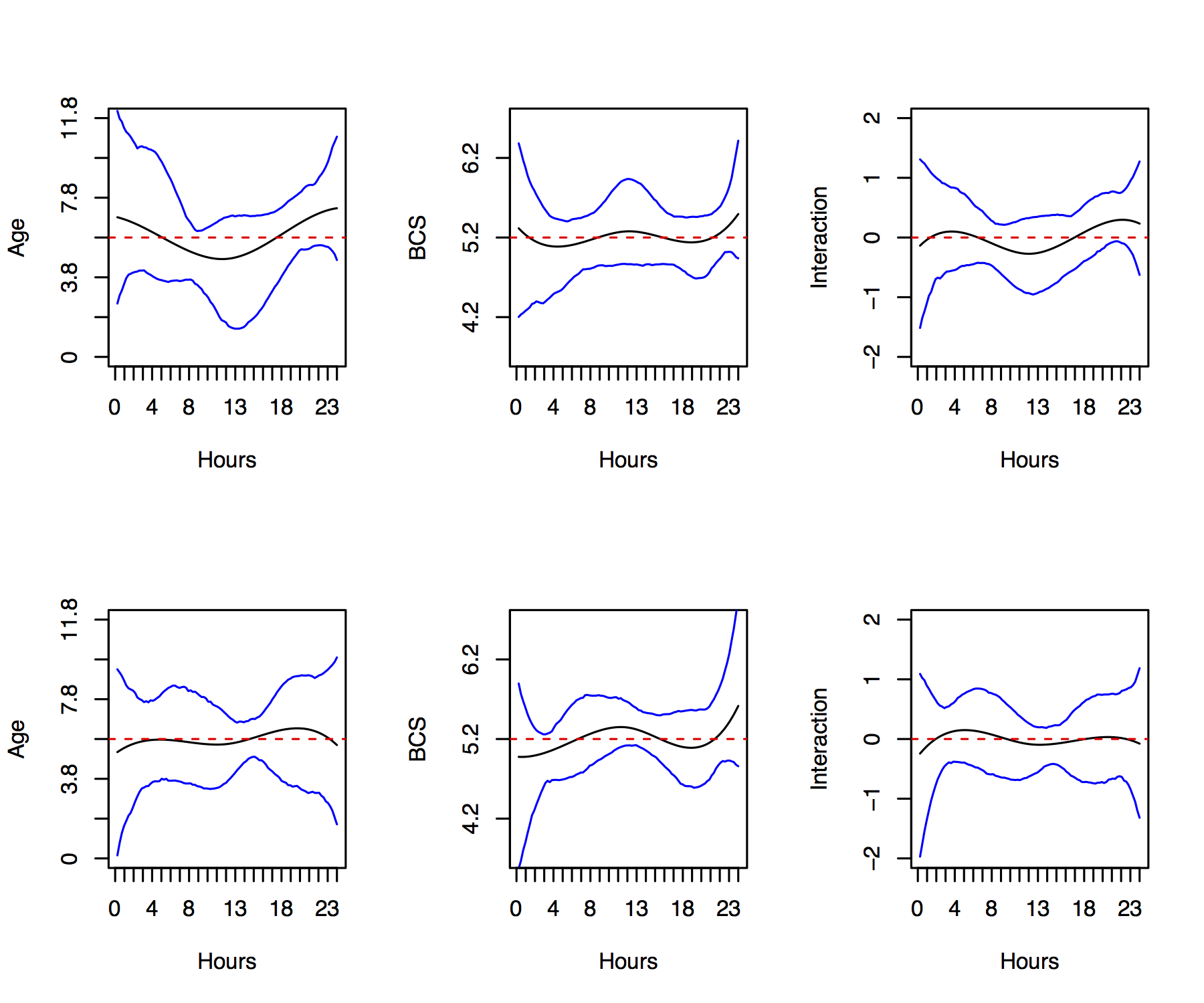

Supplement: S4 Fig — Depicted are the smooth effects of Age (years, left panels), BCS (middle panels) and AGE*BCS (standard deviations away from the mean, right panels) on the intensity of Normal cats, when model (1) is assumed. Results are shown for weekends in the top row and weekdays in the bottom row, with functional coefficients in black, 95% confidence intervals in blue, and zero demarcated in red. (TIFF) [file pone.0169576.s004.tiff]

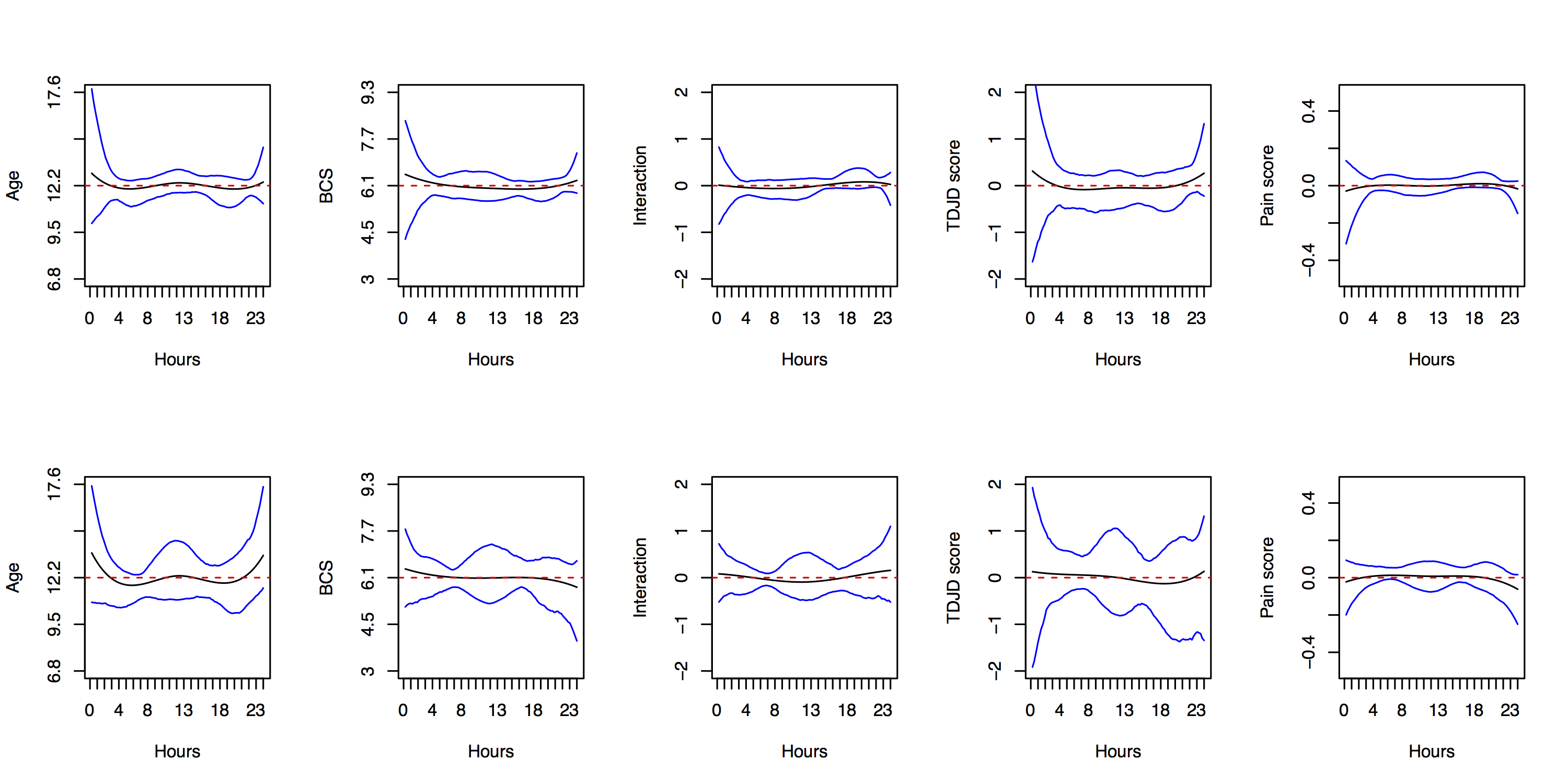

Supplement: S5 Fig — Depicted are the smooth effects of Age (years), BCS, AGE*BCS (standard deviations away from the mean), standardized TDJD score and TPain score on the intensity of DJD cats, when model (2) is assumed. Results are shown for weekends in the top row and weekdays in the bottom row, with functional coefficients in black, 95% confidence intervals in blue, and zero demarcated in red. (TIFF) [file pone.0169576.s005.tiff]
